# Supplementary material for: Convergent network effects along the axis of gene expression during prostate cancer progression
Source: Genome Biol. 2020 Dec 14;21:302. doi: 10.1186/s13059-020-02188-9 (PMC7737297; doi:10.1186/s13059-020-02188-9)
Supplement: Supplementary file 8 — Additional file 8: Review history. [file 13059_2020_2188_MOESM8_ESM.docx]

Review History

**First round of review**

**Reviewer 1**

**Are you able to assess all statistics in the manuscript, including the appropriateness of statistical tests used?** Yes, and I have assessed the statistics in my report.

**Comments to author:**

The study by Charmpi et al. presents a systematic analysis of a multi-omics dataset of 105 prostate cancer samples and 39 patients covering copy number alterations, RNA-seq, exome sequencing and proteomics. The profiled cancer samples are coupled with matched benign prostatic hyperplasia as controls which makes this dataset particularly interesting. The authors then perform a network-based analysis of the multi-omics data to quantify the changes from genomics through RNA to proteins to understand the molecular events differentiating indolent and aggressive tumors through the central dogma. The dataset itself is a valuable resource for the prostate cancer research community and the network analysis is potentially quite interesting as well. However, the manuscript is long and complex and has some ad hoc aspects that make the reader question the overall story and conclusions. Some shortening and streamlining may be in place to prioritize main findings and prune branches not essential to final conclusions. This would make the overall study an easier read. Some specific comments are listed below.

1. How many genes/proteins are reliably quantified across all the platforms, in particular RNA-seq and proteins with reasonably high non-zero transcript/peptide counts? Some known prostate cancer genes may be filtered due to low detection resolution in some of these platforms.

2. The reader might want to know about known prostate cancer driver genes, however curiously few are discussed in the manuscript. Examples include SPOP, FOXA1, ERG, AR, TP53, PTEN, etc. that have been found in various genomics and other omics studies to date. Are any of these found in the networks they discuss? Part of the problem may be that the authors chose to exclude SNV/exome sequencing data from some of their analyses and some of these genes are driven by SNVs.

3. The analysis of tumor grade centroids and individual samples with four distance measures (line 180 onwards) seems ad hoc and does not invite confidence, partly because of the conflicting results of the different distance metrics. The sample numbers are not large here (39 patients in four grade groups) and the it is not immediately clear whether the results are statistical noise. Are the distance calculations coupled with significance analysis and appropriate multiple-testing corrections?

4. The STRING database was used for finding the molecular networks distinguishing high-grade and low-grade tumors. This is potentially problematic and circular because the edges in the STRING database are partially based on computational predictions using third-party data, rather than primary molecular interactions. Consequently they report a very large network (10,000 proteins, 100,000 edges). Alternatively or in addition, they could use a true molecular interaction network (such as a physical protein-protein interaction network, or a curated pathway network, such as those in BioGrid and Reactome, respectively).

5. A similar problem concerns the "independently inferred general transcription regulatory network (bioRxiv)" (developed by some coauthors based on imputation across a variety of single-cell RNAseq data). they use this unpublished data to find trans-regulatory interactions of CNA-regions and mRNA changes. First, I am not convinced of the concept of a generic transcription regulatory network as these interactions are probably very tissue-specific and cancer programs differ from those of healthy tissues. Second, computational predictions based on computational predictions can cause unexpected biases and poor interpretability. Third, a subnetwork with regulators at most two edges away from the target genes seems like a permissive search space that has potential to explain a variety of (if not any) effects. As an alternative, the authors could consider a molecularly based regulatory network, such as abundant ChIP seq data available in ENCODE and elsewhere, to find putative transcriptional regulators. Although not admittedly noiseless, these data may provide a complementary validation approach.

6. Besides specific CNAs associating with patient survival, overall copy number alteration burden (percent genome altered) has been also linked with prostate cancer prognosis. Similarly, patient age and tumor grade are presumably associated with patient survival, and other variables. These make up potential confounding effects of the survival associations reported in this study. Are the reported survival analyses more significant than baseline models of overall copy number burden, patient age, tumor stage, etc? The authors may want to use formal Cox proportional-hazards models with covariates to test their survival associations.

**Reviewer 2**

**Are you able to assess all statistics in the manuscript, including the appropriateness of statistical tests used?** Yes, and I have assessed the statistics in my report.

**Comments to author:**

Charmpi et al study the genome, proteome and transcriptome of 39 men with localized PCa using a multi-sampling strategy. Although some studies have been performed using a similar proteogenomic setup, there are certainly many open questions and the study setup is of general interest. The study makes some interesting observations but is characterised by too many claims without clear supportive evidence. I do not agree that their data, in the current format, support the conclusion of "remarkably convergent alterations across tumor sites" (line 54) (see my comments below).

Overall, I found the main manuscript as well as Methods section fairly difficult to read. Simple concepts are over-complicated by inventing new terms (eg number of mutated genes is called 'molecular perturbation score').

General comments

- Proteomic profiling has been performed on a larger cohort (Sinha et al, 2019) that covered aspects of RNA and protein analyses. None of these findings were assessed and compared in this study. In the Sinha et al, they found CNAs to be modestly better correlated with protein than mRNA abundance. This should be discussed and compared in the current work.

- In general, it was difficult to trace which samples they used in the analyses. Inter-tumor analyses should be based on unique events only. For example, comparison between RNA and protein (eg Fig S6) - are they excluding recurrent, identical events? The same copy number event, eg deletion, in two adjacent tumor foci should not be counted twice.

- "This consistency with previously published results confirmed the previous work". It is up to the reader to plough trough the long lists of supp tables and figures to check the validity of the somatic alterations. I would like to see some numbers and comparison to the published literature on larger cohorts of PCa. For example, (Figure S2: "statistically significant point mutations in genes.") What sort of significance testing were done here? The study finds surprisingly low recurrence of some of the most recurrently mutated genes (eg FOXA1, SPOP, TP53) but instead typical false positive significant genes such as TTN (large gene).

- Line 148-149 "…several transcripts/proteins that had previously been suggested as PCa biomarker"… This has to be expanded - was a proper comparison to the literature performed? What is the rate of common CNAs and fusion genes such as ERG?

- For Fig S3, the authors need to label the individual chromosomes to evaluate the SCNAs.

- (Line 166-) How was the correlation between CNA, RNA and protein performed, using a linear model? I could not find details in the Methods - eg results in Fig S6. How are results explained showing deletions that are associated with increase in protein expression and vice versa? I am concerned that this could be due to technical issues on not reflect biology - ie measurements near detection level etc.

- The finding that proteins in complexes appear to be less affected by CNAs is to some extent expected but nevertheless interesting. The authors should perform a permutation test to control for any biases in the two set (complex vs non-complex proteins).

- The questions asked and conclusions derived from the "Inter-patient heterogeneity decreases along protein biosynthesis" section is unfortunately not clear. We know that high and low Gleason group tumors are vastly different at the molecular and pathology level. This is certainly not a new observation and has been shown many times (which is to some extent also cited in the manuscript).

- It is not clear to me what else can be learned from the "reference molecular signature" - which, as I read it, is comparing the mean fold-change from Figure 2 data. The authors correlate the perturbation for each sample to the average of the Gleason group it belongs to, another metric for variance. CNA and protein measurements correlate positively and RNA anti-correlate. It is noted that euclidian and Manhattan distance show that high-grade tumors have larger differences. Unless better explained and interpreted, I would remove this.

- Line 205: "To further corroborate the notion of common endpoints" - I dont see common endpoints but rather a potentially interesting but difficult to explain variation with increasing Gleason grade.

- The focus on the 20 most highly expressed genes appear somewhat arbitrary and not statistically motivated. For example, line 208 ""we observed that for some of these top 20 proteins"… and "in most cases" (line 212) this might be true and reflect biology, but it is rather anecdotal without any statistical assessment. For example, are these observations more prevalent in the top (20) differentially unregulated proteins compared to random (n x 20 sampled) proteins with similar absolute expression?

- Line 199: This is hypothesised to be due to "…proteins commonly affected in their abundance by oncogenic alteration". This hypothesis should be tested statistically and not just highlighting 3 genes, KLK3 being one of them.

- Analyses of trans-effects is potentially interesting. In the next section, line 237, "in most cases we observed an agreement". Is this a significant enrichment or an observation? Again, the choice if the 20 most unregulated proteins should be compared to a random set of proteins with similar absolute expression level. For the putative trans-factors, is there any orthogonal evidence for these factors as trans-factors, eg TFBS ( for those proteins with transcription factor function), which might lend support to a functional role?

- Line 260, the results do not support the statement of different patients converging on on common protein endpoints (apart from what is already known). For example, I was surprised not to find a network involving ETS and the androgen receptor, a common and recurrent transcriptional pathway deregulated in almost all PCa. This might be in the supp tables. If so, it should be highlighted.

- The finding of Network Component 1 is interesting. How many of the tumors could be reconciled with a significant enrichment of this network - was there examples of consistent activation of the network using slightly different genes in neighbouring regions of the same tumor? How does it compare to the study from Sinha et al, 2019?

**Authors Response**

**Point-by-point responses to the reviewers’ comments:**

Reviewer #1

*The study by Charmpi et al. presents a systematic analysis of a multi-omics dataset of 105 prostate cancer samples and 39 patients covering copy number alterations, RNA-seq, exome sequencing and proteomics. The profiled cancer samples are coupled with matched benign prostatic hyperplasia as controls which makes this dataset particularly interesting. The authors then perform a network-based analysis of the multi-omics data to quantify the changes from genomics through RNA to proteins to understand the molecular events differentiating indolent and aggressive tumors through the central dogma. The dataset itself is a valuable resource for the prostate cancer research community and the network analysis is potentially quite interesting as well. However, the manuscript is long and complex and has some ad hoc aspects that make the reader question the overall story and conclusions. Some shortening and streamlining may be in place to prioritize main findings and prune branches not essential to final conclusions. This would make the overall study an easier read. Some specific comments are listed below.*

We very much thank Reviewer #1 for constructive feedback. As suggested, we have removed an entire section and the corresponding figure, which helped to greatly streamline the analysis and focus it more on the key conclusions.

*1. How many genes/proteins are reliably quantified across all the platforms, in particular RNA-seq and proteins with reasonably high non-zero transcript/peptide counts? Some known prostate cancer genes may be filtered due to low detection resolution in some of these platforms.*

We have measured 17,881 genes for point mutations, 17,868 genes for CNAs, 14,281 genes on the mRNA layer and 2,371 proteins. In order to achieve high data quality, it is true that we ‘lose’ some known prostate cancer genes due to our filtering criteria especially in the proteomics dataset (see also below, e.g. we have no protein data for any of the six genes mentioned below).

*2. The reader might want to know about known prostate cancer driver genes, however curiously few are discussed in the manuscript. Examples include SPOP, FOXA1, ERG, AR, TP53, PTEN, etc. that have been found in various genomics and other omics studies to date. Are any of these found in the networks they discuss? Part of the problem may be that the authors chose to exclude SNV/exome sequencing data from some of their analyses and some of these genes are driven by SNVs.*

We already had some discussion of well-established PCa events in the manuscript, especially in the supplementary text. However, we understand that this section had to be expanded. We therefore extended the discussion of known driver events in greater detail in the revised manuscript (pages 5, 6). It now reads as follows:

“In agreement with prior reports, we observed relatively few recurrent point mutations across patients (Additional file 1: Fig. S2, Additional file 3: Table S2), but substantial CNAs

(Additional file 1: Figs. S3 and S4, Additional file 4: Table S3). Mutations in SPOP, FOXA1, and MED12 reported in independent cohorts [4-9] were confirmed in this cohort. In total, 1,110 genes showed copy number gains in at least five samples or copy number losses in at least five samples (see Additional file 1: Supplementary Text for details). Additional file 1: Fig. S4 shows the CNA status of signature genes representing known areas of recurrent CNAs in PCa- split into fusion-partner and non-fusion-partner genes- for instance loss of PTEN and gain of MYC in high-grade PCa [29]. Likewise, our data confirmed the differential expression of several transcripts/proteins that had previously been suggested as PCa biomarkers or which are known oncogenes in other tumor types (Additional file 1: Supplementary Text and Fig. S5, Additional file 5: Table S4, Additional file 6: Table S5). We further identified somatic fusions from the RNA-seq data. A large fraction of the tumors harbored ETS family gene fusions, which are frequently detected in PCa [8, 9, 11]. ETS fusions were mutually exclusive and appeared in tumors from all grade groups (Additional file 1: Fig. S6; Additional file 5: Table S4). This consistency with previously published results confirmed the quality of our data and motivated us to go beyond previous work by performing a network-based multi-omics multi-gene analysis.”

See also our response to point 3 of Reviewer #2 below.

Regarding the network analysis: the purpose of the network analysis was to find sub networks distinguishing high-grade from low-grade tumors and which were affected at all three layers: DNA, RNA and protein. However, the driver mutations mentioned by the reviewer are often present in low- and high-grade tumors, because they often initiate the neoplastic transformation and therefore do not help classifying high-grade tumors. One very clear example in our data was ERG which was strongly up-regulated across most mRNA tumor samples (47 out of 63 samples with log2FC > 1) independent of their grading. This particular analysis focuses on network effects at the DNA, RNA and protein layers in order to improve our understanding of the progression of effects. However, many oncogenic events do not display effects at all three layers. (For example, the ACP3/ACPP down regulation that we discuss elsewhere in the manuscript is caused through mutations in other genes.)

*3. The analysis of tumor grade centroids and individual samples with four distance measures (line 180 onwards) seems ad hoc and does not invite confidence, partly because of the conflicting results of the different distance metrics. The sample numbers are not large here (39 patients in four grade groups) and the it is not immediately clear whether the results are statistical noise. Are the distance calculations coupled with significance analysis and appropriate multiple-testing corrections?*

Since it is hard to convey the message of the section using the different distance measures (as pointed out by both reviewers), we have decided to remove that part. We hope that this will make the study easier to read.

See also points 8 & 9 of Reviewer #2 below.

*4. The STRING database was used for finding the molecular networks distinguishing high grade and low-grade tumors. This is potentially problematic and circular because the edges in the STRING database are partially based on computational predictions using third-party data, rather than primary molecular interactions. Consequently they report a very large network (10,000 proteins, 100,000 edges). Alternatively or in addition, they could use a true molecular interaction network (such as a physical protein-protein interaction network, or a curated pathway network, such as those in BioGrid and Reactome, respectively).*

We fully understand the concerns of the reviewer that edges in these interaction databases might occasionally be questionable. However, we do not see circularity, as the data used for the STRING database and our own data are strictly independent. (Our data was not available to STRING.) The additional sources mentioned by the reviewer already serve as input for STRING (i.e. they are part of it). STRING allows filtering edges based on diverse criteria, including filtering for experimental evidence. In order to address the reviewer’s concern we have repeated the analysis as described in the manuscript using only the information from the experimental and database channels (in STRING ‘database’ represents imports from the databases mentioned by the reviewer). The figure below displays the connected components of the resulting up-regulated sub-network. Those removed edges were relatively few, as mammalian organisms (including humans) probably represent the best-studied biological systems. As a consequence, the filtering did not significantly change our conclusions: we still capture most members of Network Component 1 while smaller or bigger parts of the other components in the figure below are part of the current results.

Further, we have checked that all the edges reported in Fig. 4a-b are supported by either experimental or database evidence no smaller than 0.348 for the up-regulated sub-network (the minimum score edge is the one connecting RUVBL1 and BANF1 supported by experimental evidence of 0.348) and 0.8 for the down-regulated sub-network. We have added this information to the figure caption.

*5. A similar problem concerns the "independently inferred general transcription regulatory network (bioRxiv)" (developed by some coauthors based on imputation across a variety of single-cell RNAseq data). they use this unpublished data to find trans-regulatory interactions of CNA-regions and mRNA changes. First, I am not convinced of the concept of a generic transcription regulatory network as these interactions are probably very tissue-specific and cancer programs differ from those of healthy tissues. Second, computational predictions based on computational predictions can cause unexpected biases and poor interpretability. Third, a subnetwork with regulators at most two edges away from the target genes seems like a permissive search space that has potential to explain a variety of (if not any) effects. As an alternative, the authors could consider a molecularly based regulatory network, such as abundant ChIP seq data available in ENCODE and elsewhere, to find putative transcriptional regulators. Although not admittedly noiseless, these data may provide a complementary validation approach.*

We fully understand the concerns of the reviewer. In the later part of the manuscript we were using the STRING network. We believe that our network is slightly more suitable for the particular purpose in this context, because it was trained to predict gene co-expression. However, it is an unpublished work. For this reason, we switched to the STRING network for this analysis as well and applied similar filtering criteria. Since STRING also includes gene co-expression as one of its ‘channels’ for predicting gene-gene relationships, it is also usable for the purpose at hand. This switch to a different network had effects on the specific neighbors that we found, however, general conclusions were not affected. In particular, also the STRING-based analysis confirmed that multiple independent CNA events likely contribute to ACPP/ACP3 downregulation.

Regarding the second point: Yes, we agree that computational predictions always need to be seen with caution. Therefore, we did not imply that the conclusions drawn from this part of the analysis were ‘proven’. We rather perceive them as guides for future research. In order to address the Reviewer’s point we have (1.) validated one of the putative ACP3 regulators using ChIP-seq data (see below) and (2.) altered the discussion to tone down our conclusions:

“A very prominent example is the indication derived from our data that the long-known PCa gene ACPP (ACP3) is down-regulated through diverse CNA events.”

I.e. we are no longer referring to this observation as a ‘discovery’, which was the term that we used in the previous manuscript.

We have addressed the third concern in two ways: first, we have limited the search space to direct STRING neighbors only. Second, we have performed statistical tests, showing that those neighbors show non-random signal in the case of ACPP (ACP3). Details on this test are provided in the manuscript.

In the case of ACP3, we observed on average significantly greater correlations between CNAs of its neighbors and the ACP3 mRNA changes, suggesting that multiple ACP3 neighbors contribute to ACP3 downregulation. To identify those potentially causal neighbors, we subsequently fitted an elastic net model (as done in the previous version of our submission).

Finally, as suggested by both reviewers, we searched for transcriptional regulators among those significant elastic net hits for ACP3 and found the transcription factor NKX3-1, which was already reported to have substantial trans effects in the Sinha et al. study. Further, we could confirm that ACP3 has previously been found to be a NKX3-1 target using ChIP-seq data. Thus, our study is now linking two known PCa genes and it provides hints towards

possible regulatory relationships among them. We have added those results to the section “Effects of distinct CNAs converge on common proteins.”

*6. Besides specific CNAs associating with patient survival, overall copy number alteration burden (percent genome altered) has been also linked with prostate cancer prognosis. Similarly, patient age and tumor grade are presumably associated with patient survival, and other variables. These make up potential confounding effects of the survival associations reported in this study. Are the reported survival analyses more significant than baseline models of overall copy number burden, patient age, tumor stage, etc? The authors may want to use formal Cox proportional-hazards models with covariates to test their survival associations.*

We thank Reviewer #1 for this comment, because it helped us to strengthen our analysis. We have fitted Cox proportional-hazards models with additional covariates, which is the current state-of-the-art as pointed out by the Reviewer. The aim of this analysis was to better understand the biology underlying PCa progression, i.e. differences between high- and low grade tumors. Thus, tumor grade is rather an output variable that we aim to predict. We therefore did not consider tumor grade as a covariate in the Cox regression. However, we did consider age and CNA burden as suggested by the reviewer.

When including age alone as an additional covariate (where available) in the model, the results show no or only minor changes. When including both age and fraction of genome altered (where available) as covariates in our model, the effect direction of Network Component 1 remained the same, but was not statistically significant anymore (*P* value = 0.055 for TCGA, mRNA and *P* value = 0.064 for MSKCC, CNA). We have added those results to page 12. We have also added the following text to the Discussion to comment on the drop of significance: “Amplification of Network Component 1 was to some extent confounded with overall CNA burden (r=0.58 (TCGA, CNA), r=0.55 (TCGA, mRNA), r=0.34 (MSKCC, CNA), r=0.16 (MSKCC, mRNA)). However, the amplifications of Network Component 1 members were highly correlated and on average above the background of CNAs. Thus, the coordinated amplification of Network Component 1 does not simply mirror overall CNA burden.”

Since the Cox regression was near significant in two independent cohorts even after accounting for mutational burden we are convinced that Network Component 1 deserves further inspection by the community and thus needs to be presented as part of our results.

Reviewer #2

*Charmpi et al study the genome, proteome and transcriptome of 39 men with localized PCa using a multi-sampling strategy. Although some studies have been performed using a similar proteogenomic setup, there are certainly many open questions and the study setup is of general interest. The study makes some interesting observations but is characterised by too many claims without clear supportive evidence. I do not agree that their data, in the current format, support the conclusion of "remarkably convergent alterations across tumor sites" (line 54) (see my comments below).*

*Overall, I found the main manuscript as well as Methods section fairly difficult to read. Simple concepts are over-complicated by inventing new terms (eg number of mutated genes is called 'molecular perturbation score').*

We thank Reviewer #2 for constructive feedback! We have tried to greatly simplify the presentation of the analysis and results, including the removal of one section. Further, we have toned down some of our conclusions and better support others.

Regarding the term ‘molecular perturbation score’: Of course, we are aware of the existing terms ‘mutational burden’ or ‘mutational load’. Those, however, only refer to the DNA layer. Here we wanted to quantify the perturbation of molecular networks at all three layers: DNA, RNA and protein. Further, we are quantifying molecular perturbations in two ways: counting

the number of events (i.e. number of mutations or differentially expressed transcripts/proteins) and second, by adding up the quantitative scores, i.e. summing the fold changes. We figured it was useful to capture the notion of these scores (i.e. their intention) in the name, i.e. the goal to quantify perturbations of molecular layers. In order to clarify this point in the manuscript we have changed the respective section as follows:

“Mutational burden is associated with PCa risk [8, 9, 11]. Hence, as a first step towards a cross-layer analysis, we asked if high-grade PCa would generally be affected by stronger alterations (compared to low-grade PCa) at the genome, transcriptome, and proteome layer [30]. For that purpose, we devised molecular perturbation scores that quantified the number of affected genes/proteins and the extent to which these genes/proteins were altered in the tumor specimens compared to their benign controls (see the ‘**Methods**’ section for details). In the case of the DNA layer these scores carry a similar meaning as established mutational burden scores. However, we wanted to capture effects at all three molecular layers measured in this study.”

*General comments*

*1. Proteomic profiling has been performed on a larger cohort (Sinha et al, 2019) that covered aspects of RNA and protein analyses. None of these findings were assessed and compared in this study. In the Sinha et al, they found CNAs to be modestly better correlated with protein than mRNA abundance. This should be discussed and compared in the current work.*

Of course, we are aware of this important study. However, the Sinha et al. study had a different research focus: whereas in the Sinha et al. study the main focus lies on differences between PCa tumor specimens and healthy tissues, our study focuses more on the molecular differences between PCa specimens of different histological grades, i.e. tumor progression.

A second limitation is the fact that Sinha et al. did not quantify protein levels in normal control samples (i.e. non-tumor samples). Therefore, we cannot easily compare fold changes between tumor and benign samples that we identified in our study to Sinha et al. Therefore, the comparability between the two studies is actually quite limited even though both quantified proteins and mRNAs in PCa.

Despite those differences in study design, we did find a number of consistent findings and no relevant inconsistencies between the two studies. In agreement with the Sinha et al. study reporting NKX3-1 to have substantial *trans* effects, we also found this gene, and could add the new finding that it is a potential regulator of ACP3, a long-known PCa gene. Further, our network propagation analysis gave ACAD9 as a hit while in the Sinha et al. study they have followed up on ACAD8, potentially suggesting that different mitochondrial members of this family are associated with PCa. Finally, the three proteins of Network Component 1 present in our study have been quantified across all samples in Sinha et al. and found to be correlated there as well. We were able to also confirm the joint activity of Network Component 1 with the microarray mRNA data.

Regarding the dependency of protein and mRNA levels on CNAs, Sinha et al. actually report that protein levels are *less* correlated with CNAs than mRNAs. Here we are citing the original publication:

“To identify *cis* effects, for each gene we compared mRNA and protein abundances between tumors with and without a CNA at that gene (6,607 genes with all three types of data available). We detected strong *cis* effects in RNA, which can be seen along the diagonal of Figure 3C as influencing ~10% of all genes (592/6,607; FDR < 0.05; t-test). These effects were present, but weaker at the protein level, with ~2% of proteins having their abundance associated to CNAs (133/6,607; Figure S4A).”

Sinha *et al.* do report slightly larger mutual information for protein-CNA relationships. That difference was however not reported to be statistically significant. (Plus, mutual information carries a different meaning than correlation.)

Thus, our results are in perfect agreement with prior literature. In order to make this clearer also in our manuscript, we have changed the respective text:

“Further, we found that mRNA fold changes (FCs) correlated more strongly with CNAs of the same genes than protein FCs (average CNA-mRNA Spearman = 0.1 and average CNA protein Spearman = 0.02). This observation is in agreement with previous work, which suggested that copy number changes are to some extent buffered at the protein level [17, 21, 32].”

*2. In general, it was difficult to trace which samples they used in the analyses. Inter-tumor analyses should be based on unique events only. For example, comparison between RNA and protein (eg Fig S6) - are they excluding recurrent, identical events? The same copy number event, eg deletion, in two adjacent tumor foci should not be counted twice.*

We are sorry for the wording that was sometimes imprecise. We have reworked the text in order to make it more clear which samples were used in which analysis mainly in the Methods section.

Our cohort includes 12 patients with 1 tumor sample and 27 patients with 2 tumor samples each. In the inter-tumor analyses, we have been using all tumor samples available at each layer. To address the Reviewer’s point, we have added the following text to the Methods section (“Network propagation/smoothing”): “It should be noted that although measurements from the same patient might not be statistically independent, we have kept them in our

analyses firstly in order to increase statistical power and secondly because not all of them correspond to clonal events as shown in **Fig. 5**. To make sure though that having two samples for some of the patients has not affected our conclusions, we have repeated the statistical testing step (one-sided Wilcoxon rank sum test) in two ways: comparing G4/5 with G1 as before, but removing the second tumor area of a patient if it belonged to the same grade group as tumor area 1 (*i.e.* removing TA2 of patients H3 and H10), and secondly comparing G4/5 with the combined (G1 and G2) group and once again removing the second tumor area of a patient if it belonged to the same grade group as his tumor area 1. *P* values resulting from these analyses were highly correlated (**Additional file 1: Fig. S9**), and we would thus consider the current conclusions to be robust.”

The analysis regarding protein-mRNA correlations among protein complex members (which is now in Fig. S7) is not affected by this issue. The reason is the following: in that part of our study we are investigating to what extent mRNA level changes impact on protein level changes. Hence, in this context it does not matter if an underlying CNA (causing the mRNA change) is clonal or not. Thus, here multiple tumor areas from the same patient can be regarded as biological replicates.

*3. "This consistency with previously published results confirmed the previous work". It is up to the reader to plough trough the long lists of supp tables and figures to check the validity of the somatic alterations. I would like to see some numbers and comparison to the published literature on larger cohorts of PCa. For example, (Figure S2: "statistically significant point mutations in genes.") What sort of significance testing were done here? The study finds surprisingly low recurrence of some of the most recurrently mutated genes (eg FOXA1, SPOP, TP53) but instead typical false positive significant genes such as TTN (large gene).*

Please also note our reply to Reviewer #1, Comments 1 & 2 above. We have compared our results to two published cohorts (Barbieri et al., 2012, and TCGA Research Network, 2015). In these cohorts, somatic mutations were also found at generally low frequencies. Remarkably, only five out of a total of 11 and 17 genes respectively overlapped between those two lists: SPOP, TP53 FOXA1, MED12 and PIK3CA. SPOP was the most frequently mutated gene there with 11 and 13% respectively while in our cohort, we found SPOP mutations in two samples, amounting to a frequency of 3%. However, we also found TP53 at around 5% and MED12 at 3%, which is in line with the frequencies reported in the aforementioned cohorts. To sum it up, given the generally low numbers of point mutations in PCa and the low frequency of recurrent mutations only a limited overlap of recurrent genes can be expected between different cohorts. This is also in line with the current belief that SNVs are not so relevant for PCa. We have added one sentence in the Supplement (Page 1) and also expanded the Fig S2 and its caption.

*4. Line 148-149 "…several transcripts/proteins that had previously been suggested as PCa biomarker"… This has to be expanded - was a proper comparison to the literature performed? What is the rate of common CNAs and fusion genes such as ERG?*

Again, please also refer to our reply to Reviewer #1, Comment 2 above. We have extended Fig S11 with copy number profiles in published PCa data to show that there is a high similarity between the CNA frequency profile in our cohort and those studies. The three datasets look highly comparable in this respect. Further, we have extended the heatmap in Fig S4 by including even more candidate driver genes and splitting them into fusion-partner

and non-fusion-partner genes. Finally, we have moved some text from the Supplement into the Main Text regarding our results on fusions from the RNA-seq data (see above and Page 6 in the MS).

*5. For Fig S3, the authors need to label the individual chromosomes to evaluate the SCNAs.* We have regenerated Fig S3 based on the Reviewer’s suggestion (see new Fig S3).

*6. (Line 166-) How was the correlation between CNA, RNA and protein performed, using a linear model? I could not find details in the Methods - eg results in Fig S6. How are results explained showing deletions that are associated with increase in protein expression and vice versa? I am concerned that this could be due to technical issues on not reflect biology - ie measurements near detection level etc.*

We have provided a more detailed description in the legend of Fig S7. We have been computing the Pearson correlation between the mRNA and corresponding protein FCs (using the 63 tumor samples available at both layers). Further, in order to address the reviewer’s concern regarding technical issues, we have computed mean signal intensities in the different groups, but did not detect any relevant differences (see also below). The apparent discrepancies mentioned by the reviewer (deletions apparently associated with greater protein levels) actually refer to non-significant mRNA and protein level changes. Therefore, we consider this simply to be noisy variation in the signals. Significant changes are outside the dashed lines in Figure S7c.

*7. The finding that proteins in complexes appear to be less affected by CNAs is to some extent expected but nevertheless interesting. The authors should perform a permutation test to control for any biases in the two set (complex vs non-complex proteins)*.

Permuting the sample labels would yield a p-value close to that from the reported t-test due to the large sample sizes. However, potential differences in the signals (protein or RNA) in the two sets could be a source for bias if for instance the stronger correlations are the result of more precise measurements in one of the two groups. In order to exclude that possibility, we computed the mean signal (mRNA and protein) of the genes in the two groups (Fig S7b) and observed that the differences between the two groups are very small. In fact, the mRNAs and proteins of complex members have on average slightly stronger signals, which would potentially reduce the technical noise. However, we observe that protein complex members exhibit weaker correlations. Thus, we believe that our conclusion is not due to any technical bias between the two gene/protein sets. We have described this in the caption of Fig S7.

*8. The questions asked and conclusions derived from the "Inter-patient heterogeneity decreases along protein biosynthesis" section is unfortunately not clear. We know that high and low Gleason group tumors are vastly different at the molecular and pathology level. This is certainly not a new observation and has been shown many times (which is to some extent also cited in the manuscript).*

Since it is hard to convey the message of the section using the different distance measures (as pointed out by both reviewers, see Reviewer 1, Comment 3 above), we have decided to remove that part. We hope that this will make the study easier to read.

*9. It is not clear to me what else can be learned from the "reference molecular signature" - which, as I read it, is comparing the mean fold-change from Figure 2 data. The authors correlate the perturbation for each sample to the average of the Gleason group it belongs to, another metric for variance. CNA and protein measurements correlate positively and RNA anti-correlate. It is noted that euclidian and Manhattan distance show that high-grade tumors have larger differences. Unless better explained and interpreted, I would remove this.*

Same as above.

*10. Line 205: "To further corroborate the notion of common endpoints" - I dont see common endpoints but rather a potentially interesting but difficult to explain variation with increasing Gleason grade.*

We have changed the introduction to the respective section in the following way to explain better the underlying notion:

“It has previously been suggested that mutations affecting different genes could impact common molecular networks if the respective gene products interact at the molecular level [36]. However, previous analyses were mostly restricted to individual molecular layers. For example, it was shown that genes mutated in different patients often cluster together in molecular interaction networks [36]. Yet, effects of these mutations on transcript and protein levels remained unexplored in this case

Previous work of Sinha and colleagues already suggested extensive *trans*-effects of CNAs on mRNA and protein levels [17]. Thus, here we aimed to systematically explore how different CNA events would impact on the level of one common protein.”

Thus, the common endpoints are proteins with frequently altered levels across our PCa cohort, whose level changes could not be explained by local CNAs. Thus, we aimed to identify *trans*-acting CNA events potentially contributing to those alterations.

*11. The focus on the 20 most highly expressed genes appear somewhat arbitrary and not statistically motivated. For example, line 208 ""we observed that for some of these top 20 proteins"… and "in most cases" (line 212) this might be true and reflect biology, but it is rather anecdotal without any statistical assessment. For example, are these observations more prevalent in the top (20) differentially unregulated proteins compared to random (n x 20 sampled) proteins with similar absolute expression?*

We agree that the number 20 is rather arbitrary. However, this analysis aimed to identify proteins with frequently modulated levels (see also our reply to the previous comment) and to illustrate one way to identify putative regulators. Thus, we simply required a sufficient number of proteins with strong concentration changes to yield examples for our analysis. A more systematic analysis distinguishing high-grade from low-grade tumors (network propagation) follows in the subsequent section. In addition, random sampling would not yield additional insight for the following reason: picking 20 proteins at random, this set will have FCs centered around 0 and having very few copy number events will be expected in this

case - but not for the top (20) most differentially regulated proteins. The following figure is trying to make this point: our choice of the top 20 most differentially expressed proteins identified proteins with FCs dramatically different from the background, whereas their local CNA patterns are almost identical to the genomic background. Therefore, there is a great chance that the protein level changes are caused by additional *trans*-effects (which by the way extends ideas of Sinha et al.).

Fig: Density plot of the observed local CNAs for 19 of the top 20 most differentially expressed proteins (one did not have CNA measurements) in all 66 tumor samples (red). Each gray curve (100 in total) corresponds to the density plot of the local CNAs of 20 proteins randomly selected across the 66 tumor samples (i.e. 20x66 values) (left). Density plot of the FCs for the top 20 proteins in all 66 tumor samples (red). Density plots in gray display the FCs of 20 proteins randomly selected across the 66 tumor samples (right). (The random sets are the same in the two plots.)

*12. Line 199: This is hypothesised to be due to "…proteins commonly affected in their abundance by oncogenic alteration". This hypothesis should be tested statistically and not just highlighting 3 genes, KLK3 being one of them.*

We have removed the section (see also Comment 10 above).

*13. Analyses of trans-effects is potentially interesting. In the next section, line 237, "in most cases we observed an agreement". Is this a significant enrichment or an observation? Again, the choice if the 20 most unregulated proteins should be compared to a random set of proteins with similar absolute expression level. For the putative trans-factors, is there any orthogonal evidence for these factors as trans-factors, eg TFBS ( for those proteins with transcription factor function), which might lend support to a functional role?*

See the last 2 paragraphs of our reply to Reviewer #1, Comment 5 above. Finally, we have improved the description of the statistics in that part.

*14. Line 260, the results do not support the statement of different patients converging on on common protein endpoints (apart from what is already known). For example, I was surprised not to find a network involving ETS and the androgen receptor, a common and recurrent transcriptional pathway deregulated in almost all PCa. This might be in the supp tables. If so, it should be highlighted.*

First, we have improved the presentation of agreements with prior work, both in the main text and the supplement. (See also Comment 2 of Reviewer #1 above.)

Indeed, ERG is deregulated in 47 out of 63 mRNA samples. However, there was no protein data for ERG. Here, we begin with the protein layer and present a way to identify potential regulators for some selected target proteins.

Further, we considerably re-worked the analysis of ACP3 (ACPP) regulators and possible CNA events convergently impacting on its levels. Both, the analysis and presentation of results has been improved to address the Reviewer’s point. See also our previous comments above for more details.

*15. The finding of Network Component 1 is interesting. How many of the tumors could be reconciled with a significant enrichment of this network - was there examples of consistent activation of the network using slightly different genes in neighbouring regions of the same tumor? How does it compare to the study from Sinha et al, 2019?*

We were able to reconcile 40 tumor samples with a significant enrichment of this network at either the CNA or RNA layer. We have made it clearer that Network Module 1 was consistently amplified in both tumor areas of some patients (especially among high grade patients; both at the CNA and RNA layers):

“Further, members of Network Component 1 were consistently amplified in both tumor areas (i.e. TA1 and TA2) of six patients (H2, H4, H5, H6, H8, and H9; **Fig. 4c**), thus establishing them as likely clonal events.“

Also, we have better highlighted those samples in Figure 4c.

Comparing to Sinha et al., was a very good idea, we thank the reviewer for that suggestion! In order to compare our findings with Sinha et al., we have used the following data from Sinha et al.: CNAs, microarray mRNA expression and protein data. We were able to confirm the joint activity of Network Component 1: there was a high correlation among its gene members at the mRNA layer and to some extent among the 5 proteins that they could detect. I.e. upregulation of one network member correlated with the upregulation of other network members in the same patient. This was an important observation, because the genes are encoded on different chromosomes and thus represent independent events. This further confirms our notion that the amplification of Network Module 1 members represents a process that is likely relevant for PCa biology. However, when generating KM curves for the Sinha et al. cohort (like those shown in Fig 4d), we did not find Network Component 1 to be predictive of survival at any layer. This result differed from our findings with all other independent cohorts (Aarhus, MSKCC, and TCGA). One possible explanation (in addition to the relatively high rate of censoring in the Sinha et al. cohort) might be the lack of clinical diversity in their cohort: the Sinha et al. study only considered intermediate-risk patients, but Network Module 1 was identified as distinguishing high-grade from low-grade tumors. Thus,

the results with the Sinha et al. cohort do not contradict our findings that we obtained from the other cohorts; their cohort may simply not be suitable for validating the predictive power of Network Module 1 for clinical outcome. We are convinced that the validation in three external cohorts (TCGA, MSKCC and Aarhus) comprising a total of 709 patients, renders Network Module 1 as an interesting candidate for future research on PCa biology.

**Second round of review**

**Reviewer 1**

The authors have done a great job addressing my earlier comments presenting new analyses and streamlining the manuscript. The key points are better emphasized while the weaker areas have been removed or reduced. The presented data look cleaner too. I had only a minor comment: since this is a proteo-genomic study, differentiating genes and proteins in the text may be helpful to the reader. Symbols in italic font denote genes and transcripts by convention while symbols in regular font denote corresponding proteins.

**Reviewer 2**

The authors have improved the manuscript and responded to most of my concerns. A few remain:

- Re my first comment: The authors need to module the statement of “remarkably convergent alterations across tumor sites” in the abstract, since the manuscript still does not support such claim. The authors even contradict the statement in the next sentence. “Considerable” would be a more appropriate statement.

- re comment 3 on missing information on recurrent mutations. They state that SPOP mutations was found “in two samples, amounting to a frequency of 3%”. Didnt they only analyse 39 patients? Was all regions considered as independent tumors? This would highly inflate any significance testing and could explain why so many likely false positive genes are found as statistically significant. In general, I would not use such a relatively underpowered cohort for driver discovery but rather use lists from better powered studies to annotate presence/absence of driver mutations.

**Authors Response**

**Point-by-point responses to the reviewers’ comments:**

Reviewer #1

The authors have done a great job addressing my earlier comments presenting new analyses and streamlining the manuscript. The key points are better emphasized while the weaker areas have been removed or reduced. The presented data look cleaner too. I had only a minor comment: since this is a proteo-genomic study, differentiating genes and proteins in the text may be helpful to the reader.

Symbols in italic font denote genes and transcripts by convention while symbols in regular font denote corresponding proteins.

*We have italicized all gene/transcript names as suggested by the reviewer.*

Reviewer #2

The authors have improved the manuscript and responded to most of my concerns. A few remain:

- Re my first comment: The authors need to module the statement of “remarkably convergent alterations across tumor sites” in the abstract, since the manuscript still does not support such claim. The authors even contradict the statement in the next sentence. “Considerable” would be a more appropriate statement.

*Thank you very much for that suggestion! We have adopted that change in the revised*

*abstract.*

- re comment 3 on missing information on recurrent mutations. They state that SPOP mutations was found “in two samples, amounting to a frequency of 3%”. Didn’t they only

analyse 39 patients? Were all regions considered as independent tumors? This would highly inflate any significance testing and could explain why so many likely false positive genes are found as statistically significant. In general, I would not use such a relatively underpowered cohort for driver discovery but rather use lists from better powered studies to annotate presence/absence of driver mutations.

*We have recomputed the frequencies as ‘percent patients’ and we have also made explicit what the percentages refer to in the manuscript. Regarding the statistical significance: there was probably some misunderstanding. The ‘statistical significance’ in Figure S2 refers to the certainty at which a mutation could be called in a given sample (based on the number of mismatch reads). It does not refer to the enrichment in our cohort. We have changed the caption of Figure S2 accordingly to make this clearer.*

*We did not use our data for the de-novo discovery of driver events. Rather, we report frequencies of mutations in our cohort and we compared those to previous publications as a sanity check.*
